# Palmistry Notes

**Published:** 1887-09-10

**Authors:** 


### AMUSEMENTS FOR CONVALESCENTS.

### PALMISTRY NOTES.

(*Continued from p.* 353.)

In a former article we dealt with the external and internal appearance of the palm, apart from its lines and the indications discoverable by certain signs. We will now proceed to study the form of the fingers and the thumb—most important items in the delineation of character by the hand, for by the length or brevity of these we judge of certain important characteristics, as well as by the smooth or knotted outline which they present. It is also necessary to note the comparative softness or hardness of the hand under examination.

Let us glance for a moment at the thumb. The *upper* or nailed phalange is the throne of will; the *second* is sacred to logic; and the *third*, or root, is dedicated to pleasure and her handmaids. The strength or weakness of a hand is naturally much influenced by the formation of the thumb. In a strong, firm character you will find the first two phalanges of almost equal length.

*The first phalange long*, indicates power of will, firmness, and—if *broad*, but not clubbed—a capacity for government; a clubbed formation shows tyranny; a *short first phalange* gives weakness and indecision of character; if *thick*, obstinacy; *thin*, alacrity in accepting the decisions of others without corresponding gratitude should those decisions prove adverse to the recipient.

*The second phalange long*, gives perception, clear reasoning powers, justice. *Short*, it indicates absence of tact, want of logic, indifference, and unreliability. The formation of the root of the thumb we shall consider later, when dealing with the mounds and lines of the hand. If the thumb turn back, it is indicative of vanity and extravagance, though with other good signs in a hand it may mean generosity and impulsiveness. Any excess of this turning back shows a tendency to exaggeration, in which generosity leads to thriftlessness and impulsiveness to rashness. A strong development of will and logic will correct this tendency, while a weak development accentuates it. We will now turn to the fingers.

*Long fingers*, with a well-proportioned palm, give dignity, capacity for control in their own and the affairs of others; humour, melancholy, sympathy, particularly with joints developed; power of analysis and deduction; obedience to established authority; language.

*Short fingers* show quickness of temper, vivacity, a tendency to jump at conclusions, impatience of control, love of society, intolerance of routine, and quickness in speech. Sometimes also fussiness, with tyranny in small matters and laxitude in greater ones.

*Thick fingers*, broad at the base, indicate love of ease and luxury. Laziness, which will be overcome more easily if the hand be hard and the fingers long, than *vice versâ*.

*Soft hands* give quickness of impression, but wanting in durability; jealousy, especially if narrow. The first impulse of soft-handed persons is to please; they like to please others and to be pleased with themselves. It is fatal to wound their vanity. They are therefore not so reliable as those with firm hands.

*Hard hands*, not stiff, but firm and strong, give sincerity, obedience to duty, activity, love of travelling, business capacity, reserve, love of outdoor sports. Hard-handed persons are more faithful in love and friendship than those whose hands are soft; more reserved, not so easily offended, but more difficult to appease, because not so easily put out. They are also generally indifferent to the impression they make on others, except in the case of those with whom they desire to stand well. Their chief characteristics are pride, as distinguished from the vanity, and faithfulness, as opposed to the caprice and inconstancy, inseparable from soft hands.

(*To be continued.*)

